# Evaluation of silk fibroin-based urinary conduits in a porcine model of urinary diversion

**DOI:** 10.3389/fbioe.2023.1100507

**Published:** 2023-01-13

**Authors:** Gokhan Gundogdu, Travis Nguyen, Seyed Hossein Hosseini Sharifi, Stephanie Starek, Kyle Costa, Clara E. Jones, David Barham, Joel Gelman, Ralph V. Clayman, Joshua R. Mauney

**Affiliations:** ^1^ Department of Urology, University of California, Irvine, Orange, CA, United States; ^2^ Department of Biomedical Engineering, University of California, Irvine, Irvine, CA, United States

**Keywords:** urinary conduit, urinary diversion, silk fibroin (SF), animal model, incontinent urinary diversion

## Abstract

**Background:** The primary strategy for urinary diversion in radical cystectomy patients involves incorporation of autologous gastrointestinal conduits into the urinary tract which leads to deleterious consequences including chronic infections and metabolic abnormalities. This report investigates the efficacy of an acellular, tubular bi-layer silk fibroin (BLSF) graft to function as an alternative urinary conduit in a porcine model of urinary diversion.

**Materials and methods:** Unilateral urinary diversion with stented BLSF conduits was executed in five adult female, Yucatan mini-swine over a 3 month period. Longitudinal imaging analyses including ultrasonography, retrograde ureteropyelography and video-endoscopy were carried out monthly. Histological, immunohistochemical (IHC), and histomorphometric assessments were performed on neoconduits at harvest.

**Results:** All animals survived until scheduled euthanasia and displayed moderate hydronephrosis (Grades 1–3) in reconstructed collecting systems over the course of the study period. Stented BLSF constructs supported formation of vascularized, retroperitoneal tubes capable of facilitating external urinary drainage. By 3 months post-operative, neoconduits contained *α*-smooth muscle actin+ and SM22α+ smooth muscle as well as uroplakin 3A+ and pan-cytokeratin + urothelium. However, the degree of tissue regeneration in neotissues was significantly lower in comparison to ureteral controls as determined by histomorphometry. In addition, neoconduit stenting was necessary to prevent stomal occlusion.

**Conclusion:** BLSF biomaterials represent emerging platforms for urinary conduit construction and may offer a functional replacement for conventional urinary diversion techniques following further optimization of mechanical properties and regenerative responses.

## Introduction

Urinary diversion with autologous gastrointestinal (GI) segments represents the primary treatment option for functional renal preservation in bladder cancer patients subjected to radical cystectomy as well as in the pediatric population afflicted with spina bifida and bladder exstrophy (Kates et al., 2015; Adamowicz et al., 2019). Several modes of urinary diversion exist including the commonly used, incontinent ileocutaneostomy which serves as a urinary conduit with exterior skin level drainage, and continent diversions such as non-orthotopic and orthotopic approaches including neobladders (Lee et al., 2014; Stein and Rubenwolf, 2014). Despite use as a front line therapy, urinary diversions are fraught with complications. Following cystectomy with urinary diversion, the early complication rate is estimated to be 50%–70%, with a 25% likelihood of readmission within 90 days, a 20% chance of intensive care unit admission, and a 3% risk of perioperative death (Dobruch et al., 2016). In addition, post-operative complications related to bowel harvesting for conduit creation include anastomosis insufficiency leading to digestive fistulae, concomitant peritonitis, and sepsis which occur in 18% of patients and required re-intervention in 50% of cases (van Hemelrijck et al., 2013). Transposition of GI segments into the urinary tract following urostomy implantation is also associated with deleterious side-effects including chronic urinary tract infections, urinary calculi, and metabolic abnormalities (Falagas and Vergidis, 2005; Okhunov et al., 2011; Stein et al., 2012). These studies highlight the significant need for the development of non-enteric, urinary diversion techniques which can overcome limitations associated with current approaches.

Over the past 15 years, tissue engineering strategies for urinary diversion have been investigated as alternatives to bowel tissue for the creation of urinary conduits (Sloff et al., 2014; Kates et al., 2015; Adamowicz et al., 2019). Scaffold designs for tissue engineered urinary conduits (TEUC) have been primarily constructed from porous, biodegradable biomaterials capable of facilitating host tissue ingrowth from ureteral anastomotic borders. The goal of these technologies is to create durable, vascularized neotissues containing contractile smooth muscle layers and a urothelial-lined lumen sufficient to promote urine peristalsis and prevent urinary extravasation, respectively (Kates et al., 2015; Adamowicz et al., 2019). TEUC composed of natural biomaterials including decellularized bladder matrices, decellularized small intestinal submucosal (SIS) scaffolds, and collagen foams, as well as synthetic polymer meshes such as polyglycolic acid (PGA), poly(lactic-co-glycolic acid) (PLGA), and polypropylene meshes have been previously explored in animal models and/or clinical studies either as acellular grafts or exogenously seeded with autologous primary or stem cell sources (Bodin et al., 2010; Basu et al., 2012; Drewa et al., 2012; Geutjes et al., 2012; Liao et al., 2013a; Liao et al., 2013b; Engberg et al., 2013; Bivalacqua et al., 2014a; Bivalacqua et al., 2014b; Sloff et al., 2016; Singh et al., 2018; Jundziłł et al., 2021; Casarin et al., 2022).

In general, preclinical assessments of cell-seeded TEUC were found to promote superior tissue regenerative responses and preserve upper urinary tract function in comparison to acellular graft configurations which routinely elicit scar tissue formation and lead to severe hydronephrosis secondary to conduit obstruction (Liao et al., 2013a; Liao et al., 2013b; Sloff et al., 2016). However, cell-seeded strategies require secondary surgeries and substantial laboratory infrastructure for cell isolation and expansion, respectively, which may limit their widespread adoption (Adamowicz et al., 2019). In the case of urologic malignancies, urinary tract-derived, primary cell populations can also be compromised by disease and therefore unsuitable for cell-seeded, construct development (Drewa et al., 2012). Moreover, first in man trials of a TEUC composed of tubular PLGA grafts seeded with adipose mesenchymal stem cell-derived, smooth muscle cells demonstrated significant adverse events including conduit stenosis and stricture formation in half of study participants (Bivalacqua et al., 2014a; Bivalacqua et al., 2014b). Given the limitations with conventional TEUC devices, we hypothesized that an acellular urinary conduit with structural, mechanical, and degradative properties sufficient to maximize host ureteral ingrowth, minimize fibrosis, and support renal function would serve as a superior candidate for urinary diversion.

Protein-based, bi-layer silk fibroin (BLSF) grafts represent an ideal platform for the construction of urinary conduits due to their high tensile strength and elasticity, low immunogenicity, and tunable biodegradability (Sack et al., 2016). These matrices can be easily sutured and utilized as sheets or tubes for urinary tract reconstruction (Affas et al., 2019; Gundogdu et al., 2021a). The structural architecture of the bi-layer matrix prevents urinary extravasation at scaffold integration sites *via* a fluid-tight film component, while an annealed porous foam layer supports surrounding tissue ingrowth (Seth et al., 2013; Tu et al., 2013). Previous studies from our laboratory have demonstrated the utility of these scaffolds for augmentation cystoplasty, urethroplasty, corporoplasty and ureteroplasty in preclinical animal models (Algarrahi et al., 2018; Affas et al., 2019; Gundogdu et al., 2021a; Gundogdu et al., 2021b). BLSF grafts have also been shown to promote formation of innervated, vascularized neotissues with functional contractile/relaxation properties and less inflammatory reactions compared to traditional decellularized matrices such as SIS scaffolds (Chung et al., 2014a; Chung et al., 2014b; Algarrahi et al., 2015). In the present study, we evaluated the efficacy of acellular, tubular BLSF grafts to function as urinary conduits in a porcine model of urinary diversion.

## Materials and methods

### Biomaterials

**FIGURE 1:**
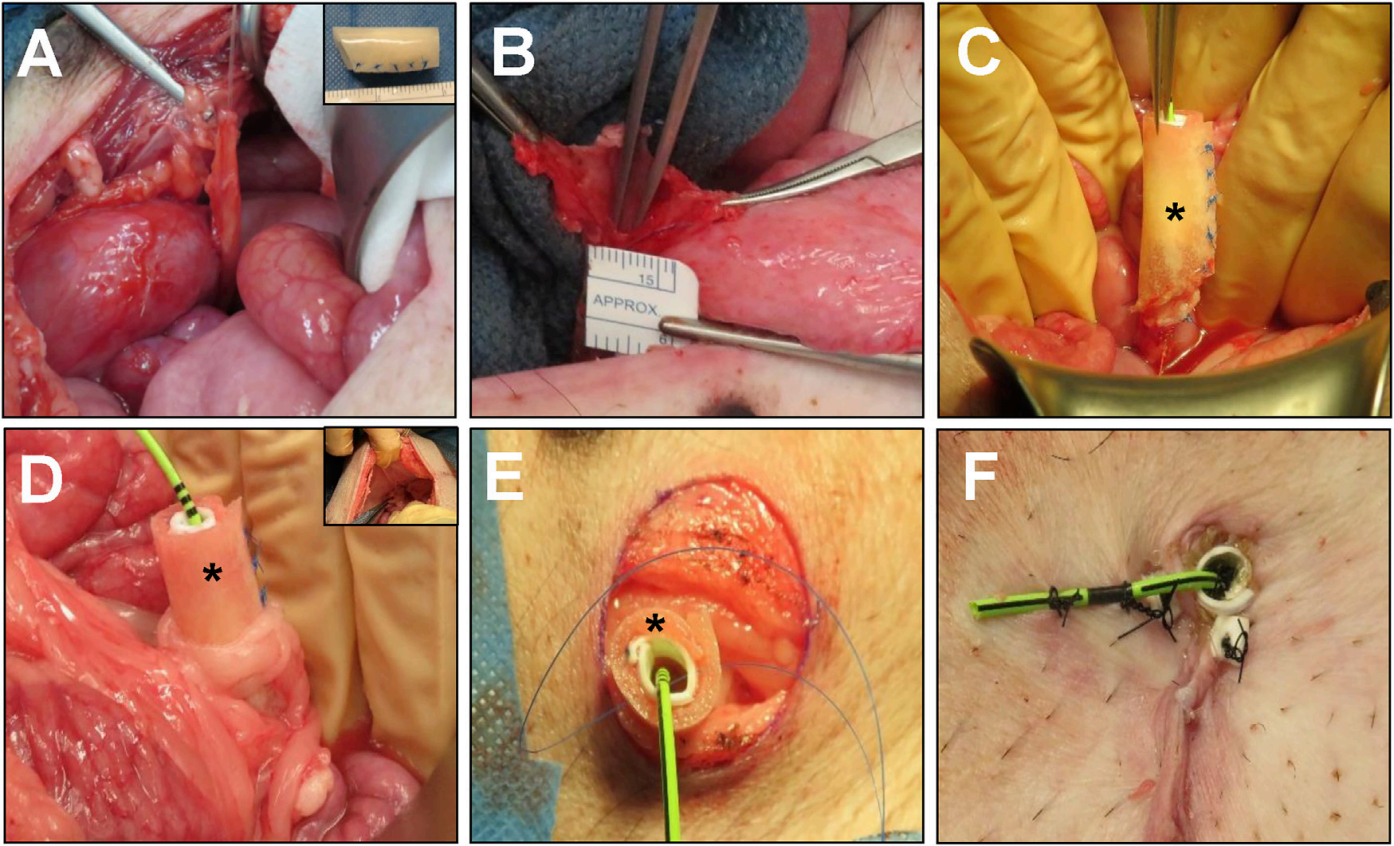
Porcine unilateral urinary diversion model. **(A)** Isolation of the right ureter with vascular supply preservation. Inset: BLSF tubular conduit. **(B)** Spatulation of the right ureter for construct implantation. **(C)** Oblique end-to-end anastomosis of BLSF conduit to the right ureter. **(D)** An omental flap was wrapped around the anastomosis and proximal half of the conduit to support *de novo* vascularization. Inset: Insertion of conduit through abdominal wall with omental wrap covering. The ureter-conduit anastomosis line was 3–5 mm away from the conduit insertion site at the abdominal wall. **(E)** Stomal creation and BLSF conduit placement at skin level. **(F)** Distal end of the BLSF construct with conduit and ureteral stent deployed and anchored to skin. (*) = BLSF tubular conduit *in situ*. BLSF, bi-layer silk fibroin scaffold.

BLSF scaffolds were constructed from aqueous silk fibroin solutions derived from *Bombyx mori* silkworm cocoons using a solvent-casting/salt-leaching procedure in combination with silk fibroin (SF) film casting as previously described (Seth et al., 2013). The mechanical and structural properties of the matrix have been reported in published studies (Seth et al., 2013). Biomaterials were sterilized with an autoclave before surgical procedures. Prior to implantation, BLSF grafts were tubularized under aseptic conditions using interrupted, non-absorbable 5–0 sutures to create a urinary conduit (inner diameter, ∼1 cm; length 3–4 cm) (Figure 1A, insert).

### Surgical procedures

Surgical, imaging and animal husbandry protocols were evaluated and approved by the University of California, Irvine Animal Care and Use Committee in accordance with protocol AUP-20–167. All animal procedures were carried out in compliance with the National Institutes of Health’s Guidelines for the Care and Use of Laboratory Animals and ARRIVE guidelines (https://arriveguidelines.org). Urinary diversion with BLSF conduits was performed in five adult female, Yucatan mini-swine (30–40 kg, ∼24 weeks of age, Premier BioSource, Ramona, CA) utilizing a uretero-cutaneous approach to create an incontinent urostomy (Figure 1). Male swine were omitted from the study to avoid incisional complications with penile anatomy during abdominal exploration and conduit formation.

Prior to surgery, animals were fasted overnight with unabated access to water. General anesthesia was induced and maintained in swine as previously described (Gundogdu et al., 2021a). Animals were then fixed in the supine position and renal sonography was performed as described below. The surgical area was scrubbed with povidone iodine and 70% ethanol three times and draped sterilely. The right ureter was accessed through a midline vertical lower abdominal incision followed by exposure of the retroperitoneum. The distal end of the right ureter was then ligated and dissected from the bladder with preservation of the vascular supply (Figure 1A) while the left ureter was left intact. The right ureter was spatulated to accommodate the circumference of the BLSF conduit (Figure 1B). Next, a 4.7 French, 24 cm double pigtail ureteral stent (Inlay Optima; BARD Inc., Covington, GA, United States) was introduced into the right ureter and the uretero-conduit anastomosis was completed using interrupted, 5–0 monofilament poliglecaprone sutures. The proximal end of the BLSF conduit was anastomosed to the spatulated ureter using an end-to-end approach in Pig 1 while an oblique end-to-end anastomosis was utilized in Pigs two to five to mitigate angulation between the conduit and the ureter (Figure 1C). In all animals, the anastomotic line was marked by 4 non-absorbable nylon sutures and two small radiopaque rings to identify the original conduit implantation area. An omental flap was wrapped around the anastomosis to support *de novo* vascularization and prevent potential anastomotic leakage (Figure 1D). Anterior-posterior, abdominal X-rays were acquired to confirm proper ureteral stent position as described below.

A conduit stoma was created at the lateral (Pig 1) or lower abdominal wall (Pigs 2–5) adjacent to the right hind leg. Briefly, a circular incision ∼15 mm in diameter was made at the skin level and abdominal wall layers were dissected to create the conduit track. The BLSF conduit was delivered through the abdominal wall defect and then sutured to surrounding tissues to prevent parastomal herniation (Figure 1E). The distal end of the conduit was adjusted to protrude beyond the skin level by 2–3 mm and was then sutured to the skin with 4–0 interrupted, monofilament poliglecaprone sutures (Figure 1F). The distal end of the double pig-tail stent was subsequently trimmed and anchored to the skin with nylon sutures to mitigate stent dislodgement. Abdominal wall layers and skin were closed separately with absorbable sutures. In Pigs two to five, a short silicone stent (inner diameter, 6 mm; length 2.5 cm) was placed down to the distal end of the BLSF conduit to prevent acute stomal stenosis. This stent was fixed to the stoma edges with two additional nylon sutures. Both ureteral and conduit stents were replaced in all swine at 1 and 2 months post-operatively or at intermediate timepoints if stent dislodgement occurred, using standard procedures (Gundogdu et al., 2021a). Briefly, an 8.5 French, 90 cm single J stent (Gyrus Medical Ltd., Wokingham, United Kingdom) was deployed into the renal pelvis using guidewire assistance and conduit stents were replaced as previously described. In addition, an 18 French, 15 mm segment of a urinary catheter (Covidien, Dublin, Ireland) was also positioned into the distal conduit and fixed to both conduit and ureteral stents to mitigate stoma stenosis.

Post-operative pain control and antibiotic regimens were executed in swine following the protocols previously described by Gundogdu and others (Gundogdu et al., 2021a). Pig 1 was maintained with a ureteral stent alone for the duration of the study and developed stomal stenosis as described below. Therefore, Pigs two to five were supported with both a silicone stomal stent and ureteral stent for the entire study period.

Longitudinal imaging analyses were carried out prior to graft implantation and at 1, 2, and 3 months post-operative to monitor urinary conduit and ureteral continuity, kidney architecture, and orientation of indwelling catheters. These assessments included video-endoscopy (ureterorenoscopy, cystoscopy), retrograde ureteropyelography (RUPG), and ultrasonography (USG) as described below. All swine were survived for a total of 3 months and then euthanized with 0.2 ml/kg pentobarbital sodium and phenytoin sodium euthanasia mixture (Euthasol; Virbac AH, Westlake, TX, United States) given intravenously. Following sacrifice, the urinary conduit as well as right (operated) and left (unoperated) ureters were harvested from the urinary tract. The urinary conduit was divided along the central axis into 4 circumferential rings (∼0.6 cm in length) including the proximal anastomosis (adjacent to the host ureter), stomal region, and two central zones of neotissues (proximal and distal conduit). Conduit and ureteral specimens were then assessed with histological, immunohistochemical (IHC), and histomorphometric analyses.

### Imaging studies

USG was executed on all animals at selected timepoints and hydronephrosis was scored using previously reported methods (Onen, 2007; Gundogdu et al., 2021a). Ureteral stent deployment and luminal conduit assessments were performed with a flexible uretero-renoscope (URS) (Flex-X2S; Karl Storz, Tuttlingen, Germany). Neotissue anastomotic borders were located with radiopaque markers placed following scaffold implantation. Images were captured with a video processor system (Image 1 HUB; Karl Storz, Tuttlingen, Germany). Contrast imaging of the conduit and urinary tract was performed by infusing 1:1 diluted iohexol contrast agent (Omniopaque 300; GE Healthcare, Milwaukee, WI, United States) through the conduit stoma orifice. The conduit and anastomosis line were evaluated with anterior-posterior, lateral and oblique images following stent removal and acquired with standard fluoroscopy methods from our published reports (Rangarajan and Somani, 2019).

### Histological, IHC, and histomorphometric analyses

Conduit (proximal anastomosis, stoma, proximal conduit, distal conduit, N = 5 animals per region) and ureteral (non-operated left and operated right ureters, N = 5 animals per region) specimens were fixed in 10% neutral-buffered formalin, dehydrated in alcohol solutions, and paraffin embedded. Five micron sections were cut and stained with Masson’s trichrome (MTS) utilizing routine histological protocols. Parallel specimens were subjected to IHC evaluations following antigen retrieval in sodium citrate buffer (10 mM, pH 6.0) and incubation in blocking buffer containing phosphate-buffered saline, 5% fetal bovine serum, 1% bovine serum albumin, and 0.3% Triton X-100 for 1 h at ∼25°C. Samples were independently incubated for 12 h at 4°C with the following primary antibodies: anti-α-smooth muscle actin (SMA) (1:200 dilution; Sigma-Aldrich, St. Louis, MO), anti-SM22α (1:200 dilution, Abcam, Cambridge, MA), anti-pan-cytokeratin (CK) (1:150 dilution; Dako, Carpinteria, CA), anti-uroplakin (UP) 3A (1:10 dilution, Fitzgerald, North Acton, MA), and anti-CD31 (1:100 dilution; Abcam). Following primary antibody incubation, samples were then probed with species-matched Alexa Fluor 647-conjugated secondary antibodies (Thermo Fisher Scientific, Waltham, MA). Nuclear counterstain was subsequently performed with 4′, 6-diamidino-2-phenyllindole (DAPI). Sample visualization was carried out with a Zeiss Axio Imager M2 model (Carl Zeiss MicroImaging, Thornwood, NY) and representative fields of interest were captured with Zen software (version 3.1). Negative controls were stained in parallel with secondary antibodies alone and generated no detectable signal above background levels.

Histomorphometric analyses (N = 5 animals per region) were performed on global 5X microscopic fields encompassing the entire circumference of the tissue specimen using published protocols (Affas et al., 2019). Imaging thresholding and area measurements were carried out with ImageJ software (version 1.47) to calculate the percentage of tissue area stained for pan-CK, *α*-SMA, and SM22α per total field area acquired. Quantitation of CD31+vessels was determined across four independent microscopic fields (20X) per specimen equally dispersed along the neotissue circumference using similar procedures and normalized to total field area to yield vascular density. Quantitation of CD31+vessel diameters in control and experimental replicates (170 ± 50 vessels per group) was performed in parallel using similar methodology.

### Statistical analysis

Quantitative data were analyzed with the Kruskal–Wallis test in combination with the *post hoc* Dunn’s test for pairwise evaluations with *p* < 0.05 defined as significant. Quantitative data were presented as mean ± standard deviation (SD).

## Results

**FIGURE 2:**
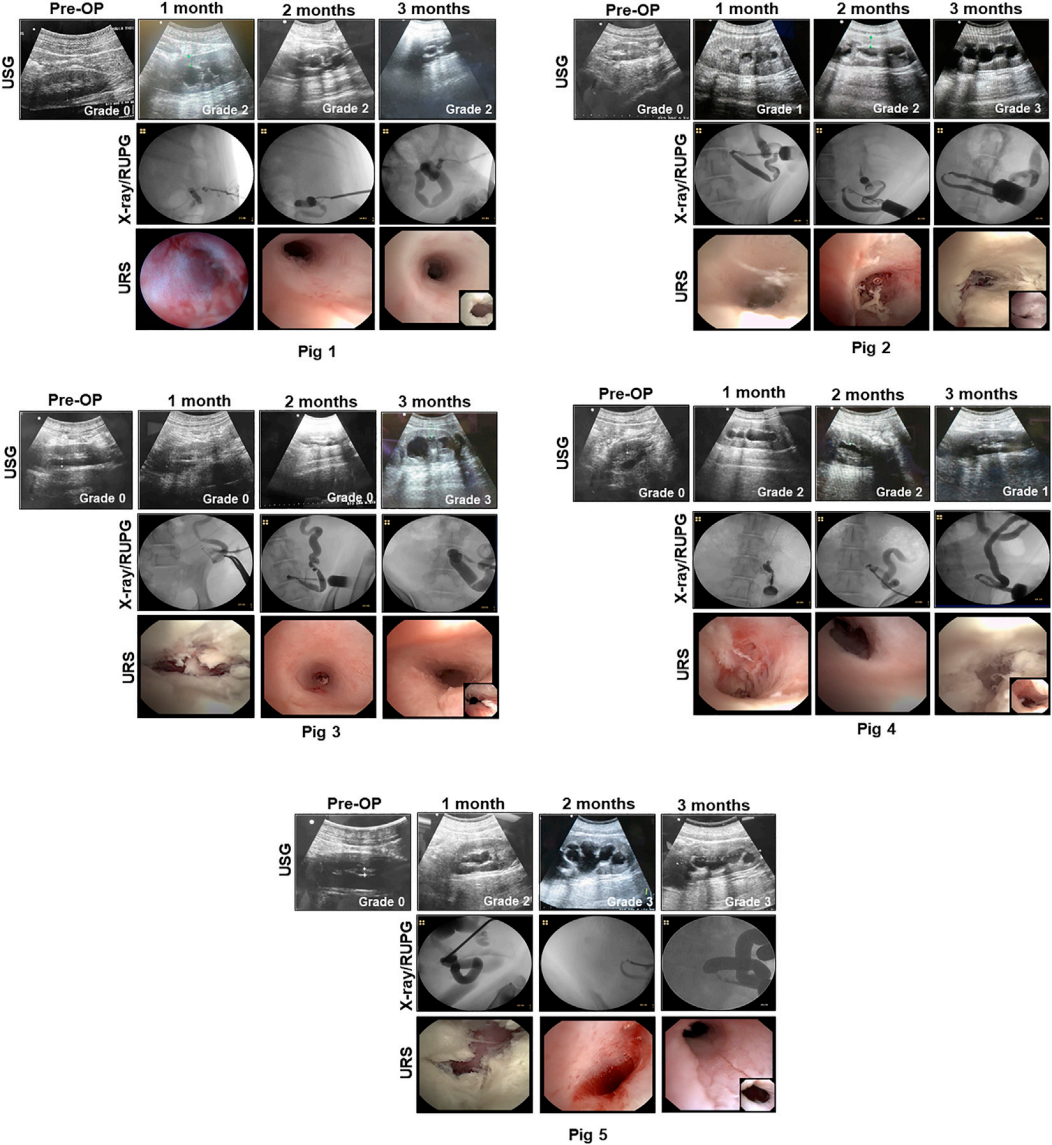
Imaging evaluations of neoconduits and upper urinary tract in reconstructed animals. Representative data for Pigs 1–5. USG (top row with hydronephrosis scores), X-ray/RUPG (second row), and URS evaluations of central neoconduits (bottom row, insets showing proximal ureteral anastomosis) were performed at various experimental timepoints. USG, ultrasonography, retrograde ureteropyelogram (RUPG), and uretero-renoscope (URS).

Unilateral incontinent urinary diversion with BLSF conduits was carried out in five swine in combination with ureteral (Pigs 1–5) and conduit (Pigs 2–5) stenting (Table 1). Stenting procedures were employed to reinforce the mechanical integrity of remodeling neotissues and alleviate potential ureteral and stomal stenosis. A unilateral approach for urinary diversion was chosen to minimize the rate of animal mortality from potential conduit failure and renal damage. Fluoroscopic, URS and USG assessments were performed throughout the study period to monitor kidney function and urinary tract continuity. (Figure 2). There were no intraoperative or immediate postoperative complications encountered during BLSF conduit implantation and all animals were successfully recovered from anesthesia and survived to scheduled euthanasia at 3 months post-operative. External urine flow from the urinary conduit was evident in all animals across all study timepoints.

**TABLE 1:** Surgical Outcomes of Urinary Diversion with BLSF conduits. Representative data from Pigs 1–5.

Animal	Urinary diversion Approach and Stent Strategy	Ultrasongraphy (Hydronephrosis Grade)				Complications and Management	terminal outcomes
		pre-op	1 month	2 months	3 months		
pig1	Incontinent urinary diversion with lateral urostomy and ureteral stenting for 3 months	0	2	2	2	Stent dislodgement and reinsertion;(post-op days 15 and 35). D iverticula at ureteral anastomosis observed at 1 month post-up	Patent tubular neotissue with no conduit or ureteral strictures. external urinary drainage detected.stomal occlusion around the ureteral stent
pig2	Incontinent urinary diversion with lower abdomnial wall urostomy and ureteral\conduit stenting for 3 months	0	1	2	3	Stent dislodgement and reinsertion:(post-op days 18,32,44,53,59,75)	Patent tubular neotissue with no conduit or ureteral strictures.perirenal cyst observed.External urinary drainage detected stomal area was 24mm^2^
pig3	Incontinent urinary diversion with lower abdomnial wall urostomy and ureteral\conduit stenting for 3 months	0	0	0	3	Stent dislodgement and reinsertion:(post-op days 59 and 75)	Patent tubular neotissue with no conduit or ureteral strictures.External urinary drainage detected.stomal area was 87mm^2^
pig4	Incontinent urinary diversion with lower abdomnial wall urostomy and ureteral\conduit stenting for 3 months	0	2	2	1	Stent dislodgement and reinsertion:(post-op days 31 and 63)	Patent tubular neotissue with no conduit or ureteral strictures.External urinary drainage detected.stomal area was 63mm^2^
pig5	Incontinent urinary diversion with lower abdomnial wall urostomy and ureteral\conduit stenting for 3 months	0	2	3	3	Stent dislodgement and reinsertion:(post-op days 38,51,and 62)	patent tubular neotissue with no conduit or ureteral strictures.External urinary drainage detected.stomal area was 75mm^2^

Pig 1 demonstrated diverticular formation at the ureteral-conduit anastomosis at 1 month post-operative and therefore an oblique anastomosis approach was adopted in subsequent animals which eliminated this problem. Ureteral stent dislodgement and reinsertion occurred 3 times over the course of the implantation period due to hind leg scratching and hence the stomal orifice was repositioned in Pigs two to five to promote stent retention. Extrusion of the BLSF conduit from the stoma occurred at 1 month post-operative in Pig 1 and URS/RUPG evaluations revealed formation of a tubular neotissue. However, stenosis of the stomal orifice was evident at this timepoint as it was fully strictured around the ureteral stent at 3 months resulting in ureteral dilation. Grade 2 hydronephrosis was observed in Pig 1 from 1 to 3 months post-operative in the reconstructed urinary tract putatively due to stomal occlusion. In Pigs two to five, additional conduit stenting approaches were utilized to ameliorate stomal stenosis including placement of a silicone stent during conduit implantation and deployment of a urinary catheter in the distal conduit from 1-3 months post-operative.

Modifications to our initial surgical approach in Pigs two to five had minimal impact on the rate of ureteral and conduit stent dislodgement. Swine required unscheduled stent reinsertion procedures 2–6 times across the study period. However, the use of primary and secondary conduit stents in this cohort did lead to substantial preservation of stomal area with mean values at 3 months post-operative reflecting 65% of the original area. BLSF conduit extrusion from the stomal orifice also occurred in Pigs two to five during 1 month stent exchanges and URS/RUPG analyses demonstrated the presence of tubular neotissues at the original graft site comparable to Pig 1 at this timepoint. Hydronephrosis and ureteral dilation were also detected in Pigs two to five from 1 to 3 months.

**FIGURE 3:**
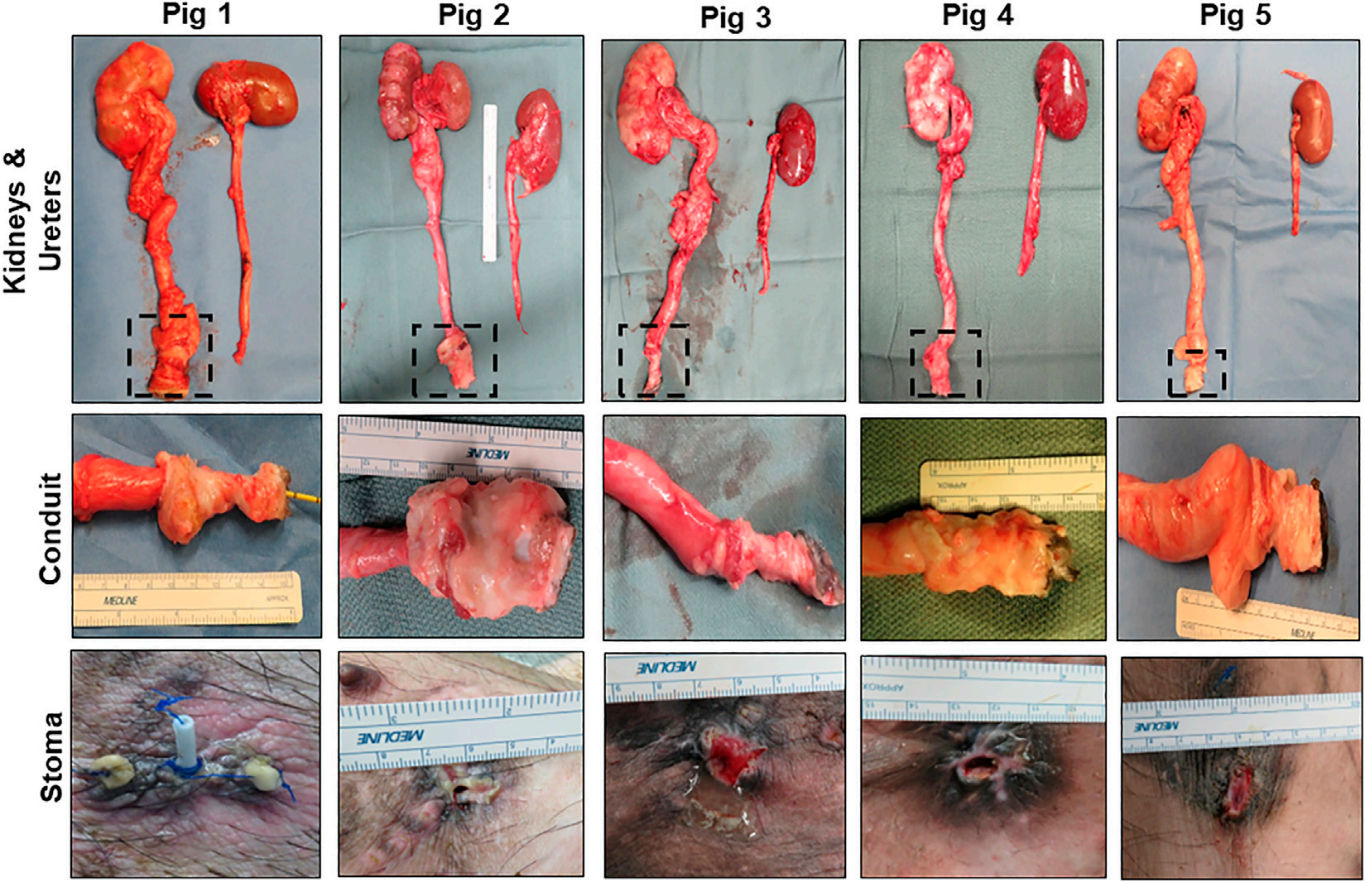
Necropsy assessments of neoconduits and reconstructed collecting systems. Top row: Photomicrographs of collecting systems and neoconduits (boxed) in Pigs one to five following 3 months of biomaterial implantation as well as parallel non-operated control ureters and kidneys. Second row: Magnified axial view of neoconduits from initial graft regions at harvest. Bottom row: Stomal orifice at 3 months post-operative.

Necropsy assessments (Figure 3) at 3 months harvest revealed host tissue ingrowth throughout the original graft site in all swine. Neotissues exhibited minimal axial contraction between the proximal/distal marking sutures and no mucosal ulceration was detected. No gross incidence of urinary stone formation or residual bulk biomaterial remnants were observed in the lumen of neotissues. Examination of the reconstructed collecting systems confirmed imaging results and revealed hydronephrosis in all animals with dilation of renal calyces and pelvises as well as hydroureters. A non-communicating perirenal cyst was also identified in Pig 2. These data were in contrast with the unoperated collecting system which demonstrated normal anatomy and no hydronephrosis.

**FIGURE 4:**
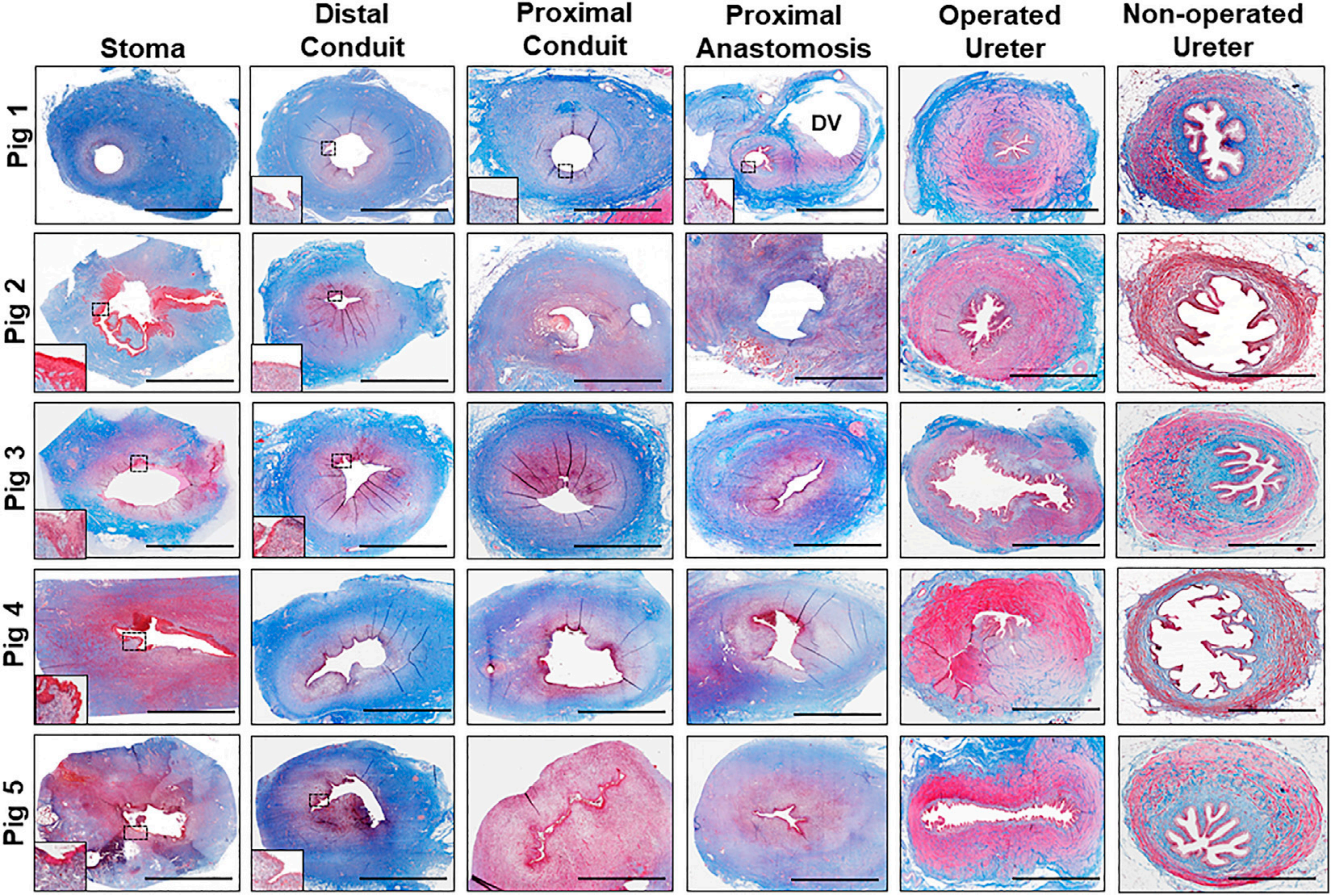
Histological evaluations of tissue regeneration in neoconduits. Photomicrographs of neoconduit cross-sections along the axial length (stoma, proximal/distal conduit, proximal anastomosis) as well as control tissues (operated and non-operated ureters) from Pigs one to five stained with Masson's trichrome. Boxed regions denote regions of de novo epithelialization with insets presenting magnified views of neoepithelia. DV denotes diverticula formation in Pig 1. Scale bars for all panels = 1 cm.

**FIGURE 5:**
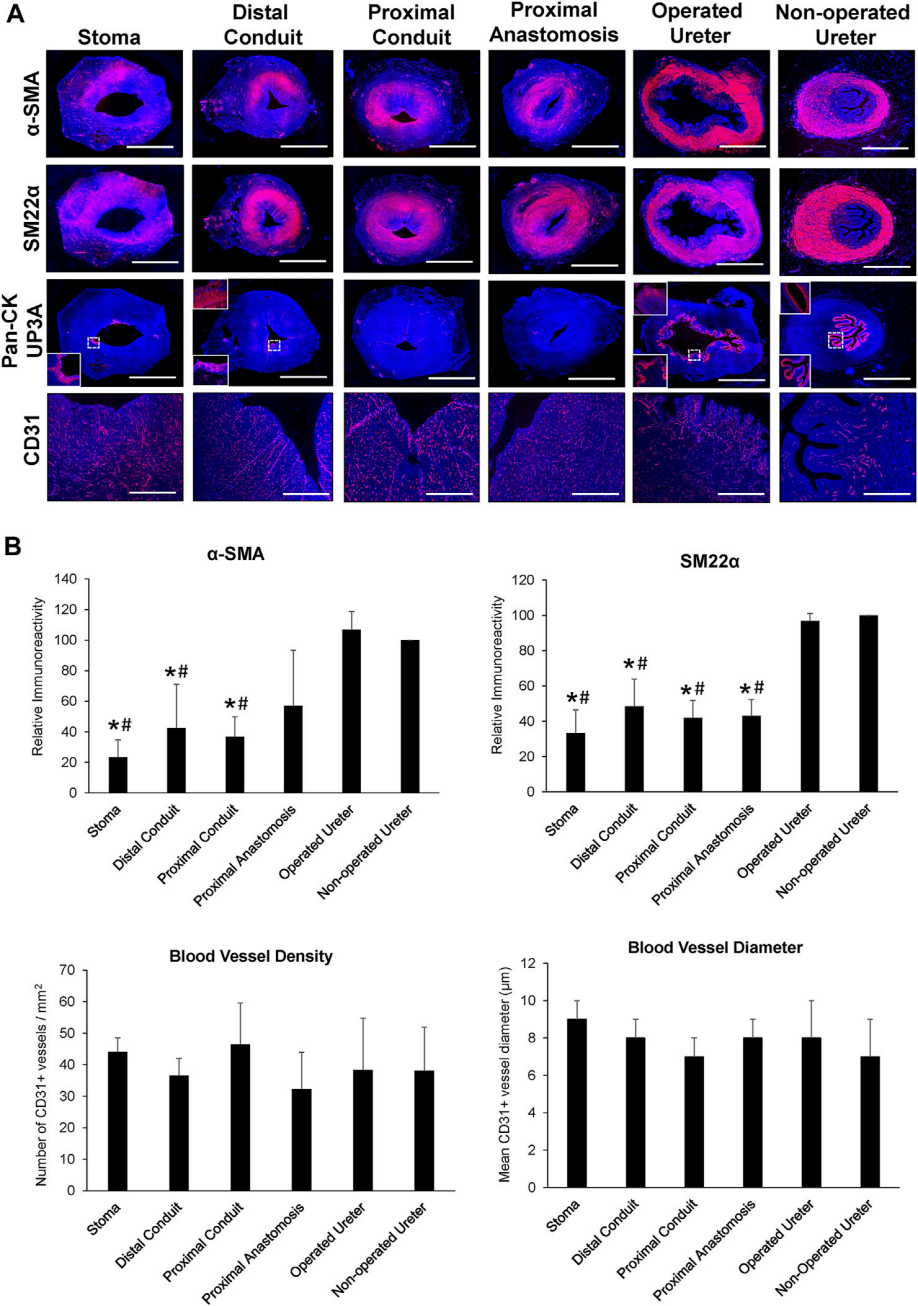
Immunohistochemical and histomorphometric analyses of neoconduit maturation. **(A)** Representative photomicrographs of selective protein expression in neoconduit regions (stoma, proximal/distal conduit, proximal anastomosis) and control tissues (operated and non-operated ureters) from Pig 3. Markers include smooth muscle contractile proteins (first and second rows: α-SMA, SM22α), epithelial proteins (third row: pan-CK, global images and bottom insets magnified from boxed area; UP3A: top insets), and vascular endothelial CD31 (fourth row). For all panels, respective marker expression is labeled in red (Alexa Fluor 594 labeling) with DAPI nuclear counterstain displayed in blue. Scale bars = 1 cm for each panel. α-SMA, a-smooth muscle actin; CK, cytokeratin; DAPI, 4′, 6-diamidino-2-phenyllindole; UP3A, uroplakin 3A. **(B)** Quantitative evaluations of markers detailed in panel A for Pigs 1–5. Data are presented as means ± SD. Results from all cohorts were analyzed with Kruskal–Wallis and post hoc Dunn's tests. P〈 0.05 relative to respective operated (*) and non-operated (#) ureteral controls.

In all five swine, global histological (MTS) evaluations of neoconduit architecture (Figure 4) demonstrated the formation of a collagenous, fibrovascular tube spanning from the ureteral anastomosis to the stoma orifice. Mononuclear inflammatory cells as well as fibroblastic cell types were dispersed throughout the *de novo* conduit wall. IHC assessments (Figure 5) revealed neoconduits contained concentric *α*-SMA + SM22α+ smooth muscle layers stretching along the entire axial length of the regenerated tissues. However, smooth muscle maturation in neotissues was underdeveloped and consisted of poorly organized, nascent bundles suggesting an ongoing state of tissue remodeling. Indeed, histomorphometric assessments revealed relative immunoreactivity of SM22α and *α*-SMA expression in stomal and distal regions of neoconduits which was significantly lower in respect to ureteral controls. Sporadic pan-CK + urothelial colonies were detected in the lumen of 4/5 neotissues scattered along the proximal and distal conduit regions. Urothelial differentiation in all neotissues was immature and incompletely stratified with weak UP3A expression in comparison to ureteral controls. Luminal infiltration of pan-CK + skin keratinocytes was also apparent in the distal regions of regenerated segments. In addition, neoconduits were highly vascularized with blood vessels lined with CD31^+^ endothelial cells apparent throughout all regions of *de novo* tissues to similar extents and with comparable diameters.

## Discussion

Current urinary diversion techniques require harvesting and incorporation of autologous GI tissues into the urinary tract which can lead to undesirable acute and chronic complications while negatively impacting upon a patient’s quality of life (Anderson and McKiernan, 2018; Rangarajan and Somani, 2019). To date, despite years of research into the utility of acellular and cell-seeded grafts as tissue engineered alternatives, there is no FDA-approved medical device for urinary diversion. Conventional decellularized tissue matrices and synthetic meshes have been explored in past reports as candidates for TEUC, however suboptimal material properties have led to chronic inflammatory reactions, scar tissue formation, stomal stenosis and conduit strictures, thus precluding clinical translation (Liao et al., 2013a; Liao et al., 2013b; Bivalacqua et al., 2014a; Bivalacqua et al., 2014b; Sloff et al., 2014). BLSF grafts have shown promise for hollow organ reconstruction in preclinical studies and demonstrated superior regenerative outcomes relative to commercial SIS matrices previously deployed as urinary conduits (Chung et al., 2014a; Chung et al., 2014b). Therefore, the goal of this study was to determine the feasibility of tubular BLSF biomaterials to serve as a urinary conduit in a porcine model of urinary diversion. Longitudinal imaging was conducted to monitor upper urinary tract function and neoconduit performance, while histological and IHC analyses were deployed to characterize the extent of tissue regeneration in reconstructed areas over the course of 3 months of implantation.

Overall, our data provide evidence that BLSF constructs in combination with ureteral/conduit stenting can support formation of vascularized, retroperitoneal tubes capable of facilitating external urinary drainage. However, the degree of tissue maturation in neoconduits at harvest at 3 months was incomplete with discontinuous smooth muscle layers and sparse epithelialization relative to ureteral controls. Moderate hydronephrosis and hydroureters were detected in all reconstructed animals and may be linked to deficiencies in neoconduit peristalsis as a result of limited smooth muscle regeneration. In addition, partial urinary tract obstruction from the presence of the conduit stent as well as aperistalsis from prolonged ureteral stenting may have also contributed to observed kidney and ureteral pathologies. Similar to our past studies in a porcine tubular ureteroplasty model, *de novo* tissue formation originated from ureteral tissue ingrowth which propagated along the exterior of the BLSF graft wall from the anastomotic border to the stomal orifice. Following bulk scaffold extrusion at 1 month, URS findings showed neomucosa spanning the entire surface of reconstructed segments suggesting neotissue expansion into implant sites led to matrix displacement and prolapse into the lumen. Comparable wound healing patterns have been reported with the use of stented PGLA-coated, PGA scaffolds as acellular urinary conduits in porcine models (Basu et al., 2012). BLSF biomaterials remained grossly intact *in vivo*, but could be easily extruded from the stomal orifice after neotissue formation potentially diminishing the risk of chronic foreign body reactions and urinary tract obstruction. Previous assessments of BLSF grafts for tubular ureteroplasty demonstrated similar degrees of degradation, however graft persistence in distal segments resulted in urinary blockage and severe renal damage (Gundogdu et al., 2021a).

Stenting of acellular urinary conduits has been reported to mitigate stomal stenosis and maintain patency of regenerated tissues by reinforcing luminal mechanical integrity during tissue remodeling (Basu et al., 2012). These results are comparable to our current findings wherein conduit stenting reduced stomal occlusion relative to untreated controls and preserved 65% of the original stomal area. Stent dislodgement occurred frequently in our animal model necessitating periodic stent exchanges to maintain stomal caliber. These manipulations may have disrupted urothelial growth and stratification in regenerated tissues due to mucosal abrasions acquired during stent deployment. Future improvements in our matrix design will focus on increasing the radial force exerted by BLSF constructs to improve graft retention and prevent stomal stenosis in the absence of stenting. Material properties including initial SF content and scaffold pore size have been implicated as significant regulators of compressive strength and stiffness in aqueous-based SF foams (Kim et al., 2005). These findings suggest that the radial strength and stiffness of the foam compartment of BLSF grafts may be enhanced to prevent stenotic events by increasing the concentration of SF used during casting or by reducing matrix pore size *via* modulation of porogen diameter. In addition, SF biomaterials have also been reported to serve as targeted drug delivery systems both *in vivo* and *in vitro* for a variety of agents including small molecules, cytokines, nucleic acids, and antibodies (Wani and Veerabhadrappa, 2018). The creation of next-generation, BLSF conduits with the capacity to stimulate smooth muscle and urothelial formation *via* controlled release of respective differentiation agonists such as bone morphogenetic protein-4 (Wang et al., 2009) and retinoic acid (Gandhi et al., 2013) may promote increased levels of functional tissue regeneration in neoconduits.

## Conclusion

This proof of concept study demonstrated that acellular, tubular BLSF grafts in tandem with stenting can promote *de novo* tissue formation and urinary diversion in a porcine model. However, at 3 months, vascularized neoconduits were composed of developmentally immature smooth muscle and urothelium relative to ureteral controls and required continuous stenting to prevent stomal occlusion. Future improvements in our original scaffold design will focus on increasing their radial mechanical integrity to maintain urostomy caliber and urinary drainage through modulations of matrix processing parameters as well as incorporation of targeted, selective drug delivery capacities to spur increased regenerative responses. In addition, validation studies of our optimized graft configuration in a preclinical model of bilateral urinary diversion are needed to simulate the clinical scenario encountered with radical cystectomy patients. In summary, BLSF biomaterials represent emerging platforms for urinary conduit construction and may offer a functional replacement for conventional urinary diversion techniques following further refinement.

## Data Availability

The original contributions presented in the study are included in the article/supplementary material, further inquiries can be directed to the corresponding author.
